# Supplementary material for: Assays for the Evaluation of the Immune Response to Marburg and Ebola Sudan Vaccination—Filovirus Animal Nonclinical Group Anti-Marburg Virus Glycoprotein Immunoglobulin G Enzyme-Linked Immunosorbent Assay and a Pseudovirion Neutralization Assay
Source: Vaccines (Basel). 2022 Jul 29;10(8):1211. doi: 10.3390/vaccines10081211 (PMC9413256; doi:10.3390/vaccines10081211)
Supplement: Supplementary file 1 [file vaccines-10-01211-s001.zip › vaccines-1783124-supplementary.pdf]

Assays for the Evaluation of the Immune Response to Marburg and Ebola Sudan Vaccination – Filovirus Ani-mal Nonclinical Group anti-Marburg Virus Glycoprotein Immunoglobulin G Enzyme-Linked Immunosorbent As-say and a Pseudovirion Neutralization Assay

Supplementary Materials

Table S1. ELISA Results for MARV and SUDV Positive Serum Samples.

Virus	Sample ID	Concentration (ELISA Units/mL)	Reagent
MARV	MK3	6761.53	RS
MARV	MK6	8754.71	RS
MARV	MK2	2841.75	QC High
MARV	MK5	2362.63	QC High
MARV	MK8	2597.80	QC High
MARV	MK1	887.50	QC Low
MARV	MK4	672.21	QC Low
SUDV	G16	1568.40	RS
SUDV	L1	4224.76	RS
SUDV	G24	622.97	High
SUDV	G26	977.81	High
SUDV	G12	320.62	Low
SUDV	G21	347.46	Low
SUDV	G23	0.00	NA
SUDV	G28	25.32	NA
SUDV	G44	69.81	NA
SUDV	G55	0.00	NA
SUDV	L2	0.00	NA

Table S2. Creation of Reference Standards and Quality Controls.

Virus	Reagent Type (Generation)	Lot Number	Composition
MARV	RS (First)	BMIMARV101	MK3 and MK6
MARV	RS (Second)	BMIMARV104	MK6 and MK9
MARV	RS (Third)	BMIMARV107	Tc Bovine
MARV	QC-High (First)	BMIMARV102	MK2, MK5, and MK8
MARV	QC-High (Second)	BMIMARV105	MK2 and MK4
MARV	QC-High (Third)	BMIMARV108	Tc Bovine
MARV	QC-Low (First)	BMIMARV103	MK1 and MK4
MARV	QC-Low (Second)	BMIMARV106	MK5 and MK8
MARV	QC-Low (Third)	BMIMARV109	Tc Bovine

SUDV	RS (First)	BMISUDV101	G16 and L1
SUDV	RS (Second)	BMISUDV104	Tc Bovine
SUDV	QC-High (First)	BMISUDV102	G24 and G26
SUDV	QC-High (Second)	BMISUDV105	Tc Bovine
SUDV	QC-Low (First)	BMISUDV103	G12 and G21
SUDV	QC-Low (Second)	BMISUDV106	Tc Bovine

Table S3. Summary of Reference Standard, Quality Control, and Negative Control Lots.

Virus Strain or Species	Reagent Type (Generation)	Lot Number	Concentration (ELISA Units/mL)	Acceptance Range (ELISA Units/mL)[a]
MARV	RS (First)	BMIMARV101	1,000[b]	NA
MARV	RS (Second)	BMIMARV104	936	NA
MARV	RS (Third)	BMIMARV107	1,058	NA
MARV	QC-High (First)	BMIMARV102	508.63	254.31 – 762.94
MARV	QC-High (Second)	BMIMARV105	464.13	280.46 – 647.80
MARV	QC-High (Third)	BMIMARV108	427.58	218.88 – 636.28
MARV	QC-Low (First)	BMIMARV103	121.48	60.74 – 182.22
MARV	QC-Low (Second)	BMIMARV106	138.21	77.08 – 199.34
MARV	QC-Low (Third)	BMIMARV109	156.09	83.75 – 228.44
SUDV	RS (First)	BMISUDV101	1,000[b]	NA
SUDV	RS (Second)	BMISUDV104	1,143	NA
SUDV	QC-High (First)	BMISUDV102	212.57	99.49 – 325.65
SUDV	QC-High (Second)	BMISUDV105	294.84	203.62 – 386.06
SUDV	QC-Low (First)	BMISUDV103	69.34	36.66 – 102.01
SUDV	QC-Low (Second)	BMISUDV106	128.13	89.55 – 166.71
Human	NC	BMI529	0.00	OD ≤ 0.20
Human	NC	BMI530	0.00	OD ≤ 0.20
Human	NC	BMI547	0.00	OD ≤ 0.20
NHP	NC	BMI298	0.00	OD ≤ 0.20
NHP	NC	BMI300	0.00	OD ≤ 0.20
NHP	NC	BMI304	0.00	OD ≤ 0.20

a – Mean ± 2 standard deviations.
b – Value was arbitrarily assigned.
NA = Not applicable

Table S4. Immune Serum Samples used to Generate QTSs.

Virus Species	Serum ID	Species	Approximate Concentration (ELISA Units/mL)
MARV	FLV1001-100023 Day 71	Human	1283.68
MARV	FLV1001-100068 Day 71	Human	2267.29
MARV	FLV1001-100074 Day 71	Human	3233.84
MARV	FLV1001-100014 Day 71	Human	4828.04
MARV	FLV1001-100073 Day 71	Human	2307.94

Virus Species	Serum ID	Species	Approximate Concentration (ELISA Units/mL)
MARV	08110.28552.D70	NHP	5160.49
MARV	08110.67181.D70	NHP	5315.32
MARV	08110.09547.D70	NHP	5351.43
MARV	08110.76302.D70	NHP	6418.43
MARV	08110.19687.D70	NHP	7469.17
SUDV	FLV1001-100027 Day 78	Human	13,143.20
SUDV	FLV1001-100108 Day 113	Human	5959.09
SUDV	FLV1001-100041 Day 78	Human	8241.61
SUDV	FLV1001-100075 Day 78	Human	4701.28
SUDV	FLV1001-100097 Day 36	Human	1848.99
SUDV	0740011.18305.D28	NHP	1299.43
SUDV	08110.19687.D28	NHP	806.77
SUDV	0740011.59335.D63	NHP	5273.21
SUDV	0740011.84569.D70	NHP	3358.48
SUDV	0740011.81800.D84	NHP	2950.13

Table S5. MARV Qualification Test Sample Dilutional Linearity Series Generation.

QTS Number	Test Specimen ID	Anti-GP IgG Concentration (ELISA Units/mL) (Before Spike)	Volume of Test Specimen (μL)	Dilution Factor (Spike)	Final Dilution Factor (Spike)	Negative Specimen Identifier (Diluent)	Volume of Negative Specimen Diluent (μL)	Total QTS Volume (μL)	Expected QTS Anti-GP IgG Concentration (ELISA Units/mL)	QTS Starting Dilution
1	FLV1001-100023 Day 71	1284	270	1:1	1:1	NA	0	270	1284	1:100
2	Same as QTS 1	1284	-	-	-	NA	-	-	1284	1:200
3	Same as QTS 1	1284	-	-	-	NA	-	-	1284	1:50
4	FLV1001-100023 Day 71	1284	125	1:2	1:2	BMI530	125	250	642	1:50
5	FLV1001-100023 Day 71	1284	24	1:5	1:5	BMI530	96	120	257	1:50
6	FLV1001-100023 Day 71	1284	30	1:8	1:8	BMI530	210	240	160	1:50
7	FLV1001-100023 Day 71	1284	10	1:16	1:16	BMI530	150	160	80	1:50
8 - Step 1	FLV1001-100023 Day 71	1284	5	1:10	NA	BMI530	45	50	NA	NA
8 - Final	8 - Step 1	128	35	1:8	1:80	BMI530	245	280	16	1:50
9 - Step 1	FLV1001-100023 Day 71	1284	3	1:10	NA	BMI530	27	30	NA	NA
9 - Final	9 - Step 1	128	12	1:10	1:100	BMI530	108	120	13	1:50
10 - Step 1	FLV1001-100023 Day 71	1284	2	1:10	NA	BMI530	18	20	NA	NA
10 - Final	10 - Step 1	128	8	1:15	1:150	BMI530	112	120	9	1:50
11	FLV1001-100068 Day 71	2267	120	1:1	1:1	NA	0	120	2267	1:100
12	FLV1001-100068 Day 71	2267	135	1:2	1:2	BMI530	135	270	1134	1:100
13	Same as QTS 12	2267	-	-	-	-	-	-	1134	1:200
14	Same as QTS 12	2267	-	-	-	-	-	-	1134	1:50
15	FLV1001-100068 Day 71	2267	30	1:4	1:4	BMI530	90	120	567	1:50
16	FLV1001-100068 Day 71	2267	30	1:8	1:8	BMI530	210	240	283	1:50
17	FLV1001-100068 Day 71	2267	15	1:16	1:16	BMI530	225	240	142	1:50
18 - Step 1	FLV1001-100068 Day 71	2267	3	1:10	NA	BMI530	27	30	NA	NA
18 - Final	18 - Step 1	227	20	1:15	1:150	BMI530	280	300	15	1:50
19 - Step 1	FLV1001-100068 Day 71	2267	6	1:15	NA	BMI530	84	90	NA	NA
19 - Final	19 - Step 1	151	12	1:20	1:300	BMI530	228	240	8	1:50
20 - Step 1	FLV1001-100068 Day 71	2267	2	1:20	NA	BMI530	38	40	NA	NA
20 - Final	20 - Step 1	113	10	1:30	1:600	BMI530	290	300	4	1:50
21	FLV1001-100074 Day 71	3234	270	1:1	1:1	NA	0	270	3234	1:200
22	Same as QTS 21	3234	-	-	-	-	-	-	3234	1:400
23	Same as QTS 21	3234	-	-	-	-	-	-	3234	1:100
24	FLV1001-100074 Day 71	3234	90	1:3	1:3	BMI530	180	270	1078	1:100

QTS Number	Test Specimen ID	Anti-GP IgG Concentration (ELISA Units/mL) (Before Spike)	Volume of Test Specimen (µL)	Dilution Factor (Spike)	Final Dilution Factor (Spike)	Negative Specimen Identifier (Diluent)	Volume of Negative Specimen Diluent (µL)	Total QTS Volume (µL)	Expected QTS Anti-GP IgG Concentration (ELISA Units/mL)	QTS Starting Dilution
25	FLV1001-100074 Day 71	3234	24	1:5	1:5	BMI530	96	120	647	1:50
26	FLV1001-100074 Day 71	3234	10	1:25	1:25	BMI530	240	250	129	1:50
27 - Step 1	FLV1001-100074 Day 71	3234	3	1:9	NA	BMI530	24	27	NA	NA
27 - Final	27 - Step 1	359	20	1:6	1:54	BMI530	100	120	60	1:50
28 - Step 1	FLV1001-100074 Day 71	3234	2	1:10	NA	BMI530	18	20	NA	NA
28 - Final	28 - Step 1	323	9	1:20	1:200	BMI530	171	180	16	1:50
29 - Step 1	FLV1001-100074 Day 71	3234	2	1:20	NA	BMI530	38	40	NA	NA
29 - Final	29 - Step 1	162	9	1:20	1:400	BMI530	171	180	8	1:50
30 - Step 1	FLV1001-100074 Day 71	3234	2	1:20	NA	BMI530	38	40	NA	NA
30 - Final	30 - Step 1	162	8	1:40	1:800	BMI530	312	320	4	1:50
31	FLV1001-100014 Day 71	4828	270	1:1	1:1	NA	0	270	4828	1:200
32	Same as QTS 31	4828	-	-	-	-	-	-	4828	1:400
33	Same as QTS 31	4828	-	-	-	-	-	-	4828	1:100
34	FLV1001-100014 Day 71	4828	40	1:3	1:3	BMI530	80	120	1609	1:100
35	FLV1001-100014 Day 71	4828	20	1:9	1:9	BMI530	160	180	536	1:50
36	FLV1001-100014 Day 71	4828	10	1:27	1:27	BMI530	260	270	179	1:50
37 - Step 1	FLV1001-100014 Day 71	4828	10	1:9	NA	BMI530	80	90	NA	NA
37 - Final	37 - Step 1	536	50	1:6	1:54	BMI530	250	300	89	1:50
38 - Step 1	FLV1001-100014 Day 71	4828	2	1:20	NA	BMI530	38	40	NA	NA
38 - Final	38 - Step 1	241	15	1:20	1:400	BMI530	285	300	12	1:50
39 - Step 1	FLV1001-100014 Day 71	4828	2	1:20	NA	BMI530	38	40	NA	NA
39 - Final	39 - Step 1	241	10	1:30	1:600	BMI530	290	300	8	1:50
40 - Step 1	FLV1001-100014 Day 71	4828	2	1:10	NA	BMI530	18	20	NA	NA
40 - Step 2	40 - Step 1	483	2	1:10	NA	BMI530	18	20	NA	NA
40 - Final	40 - Step 2	48	25	1:12	1:1200	BMI530	275	300	4	1:50
41	FLV1001-100073 Day 71	2308	250	1:1	1:1	NA	0	250	2308	1:300
42	FLV1001-100073 Day 71	2308	150	1:2	1:2	BMI530	150	300	1154	1:100
43	Same as QTS 42	2308	-	-	-	-	-	-	1154	1:200
44	Same as QTS 42	2308	-	-	-	-	-	-	1154	1:50
45	FLV1001-100073 Day 71	2308	30	1:4	1:4	BMI530	90	120	577	1:50
46	FLV1001-100073 Day 71	2308	30	1:8	1:8	BMI530	210	240	288	1:50

QTS Number	Test Specimen ID	Anti-GP IgG Concentration (ELISA Units/mL) (Before Spike)	Volume of Test Specimen (µL)	Dilution Factor (Spike)	Final Dilution Factor (Spike)	Negative Specimen Identifier (Diluent)	Volume of Negative Specimen Diluent (µL)	Total QTS Volume (µL)	Expected QTS Anti-GP IgG Concentration (ELISA Units/mL)	QTS Starting Dilution
47	FLV1001-100073 Day 71	2308	10	1:16	1:16	BMI530	150	160	144	1:50
48 - Step 1	FLV1001-100073 Day 71	2308	3	1:10	NA	BMI530	27	30	NA	NA
48 - Final	48 - Step 1	231	15	1:20	1:200	BMI530	285	300	12	1:50
49 - Step 1	FLV1001-100073 Day 71	2308	2	1:10	NA	BMI530	18	20	NA	NA
49 - Final	49 - Step 1	231	6	1:30	1:300	BMI530	174	180	8	1:50
50 - Step 1	FLV1001-100073 Day 71	2308	2	1:20	NA	BMI530	38	40	NA	NA
50 - Final	50 - Step 1	115	4	1:40	1:800	BMI530	156	160	3	1:50
51	08110.28552.D70	5160	120	1:1	1:1	NA	0	120	5160	1:600
52	08110.28552.D70	5160	60	1:8	1:8	BMI304	420	480	645	1:100
53	Same as QTS 52	5160	-	-	-	-	-	-	645	1:200
54	Same as QTS 52	5160	-	-	-	-	-	-	645	1:50
55	08110.28552.D70	5160	6	1:20	1:20	BMI304	114	120	258	1:50
56	08110.28552.D70	5160	12	1:40	1:40	BMI304	468	480	129	1:50
57	08110.28552.D70	5160	1.6	1:100	1:100	BMI304	158.4	160	52	1:50
58 - Step 1	08110.28552.D70	5160	4	1:25	NA	BMI304	96	100	NA	NA
58 - Final	58 - Step 1	206	48	1:10	1:250	BMI304	432	480	21	1:50
59 - Step 1	08110.28552.D70	5160	3	1:20	NA	BMI304	57	60	NA	NA
59 - Final	59 - Step 1	258	6	1:20	1:400	BMI304	114	120	13	1:50
60 - Step 1	08110.28552.D70	5160	2	1:25	NA	BMI304	48	50	NA	NA
60 - Final	60 - Step 1	206	3	1:40	1:1000	BMI304	117	120	5	1:50
61	08110.67181.D70	5315	270	1:1	NA	NA	0	270	5315	1:700
62	Same as QTS 61	5315	-	-	-	-	-	-	5315	1:1400
63	Same as QTS 61	5315	-	-	-	-	-	-	5315	1:350
64	08110.67181.D70	5315	15	1:8	1:8	BMI304	105	120	664	1:50
65	08110.67181.D70	5315	6	1:20	1:20	BMI304	114	120	266	1:50
66	08110.67181.D70	5315	6	1:40	1:40	BMI304	234	240	133	1:50
67	08110.67181.D70	5315	2	1:80	1:80	BMI304	158	160	66	1:50
68 - Step 1	08110.67181.D70	5315	2	1:10	NA	BMI304	18	20	NA	NA
68 - Final	68 - Step 1	532	6	1:20	1:200	BMI304	114	120	27	1:50
69 - Step 1	08110.67181.D70	5315	2	1:20	NA	BMI304	38	40	NA	NA
69 - Final	69 - Step 1	266	12	1:20	1:400	BMI304	228	240	13	1:50

QTS Number	Test Specimen ID	Anti-GP IgG Concentration (ELISA Units/mL) (Before Spike)	Volume of Test Specimen (µL)	Dilution Factor (Spike)	Final Dilution Factor (Spike)	Negative Specimen Identifier (Diluent)	Volume of Negative Specimen Diluent (µL)	Total QTS Volume (µL)	Expected QTS Anti-GP IgG Concentration (ELISA Units/mL)	QTS Starting Dilution
70 - Step 1	08110.67181.D70	5315	2	1:20	NA	BMI304	38	40	NA	NA
70 - Final	70 - Step 1	266	8	1:40	1:800	BMI304	312	320	7	1:50
71	08110.09547.D70	5351	120	1:1	1:1	NA	0	120	5351	1:700
72	08110.09547.D70	5351	120	1:4	1:4	BMI304	360	480	1338	1:100
73	Same as QTS 72	5351	-	-	-	-	-	-	1338	1:200
74	Same as QTS 72	5351	-	-	-	-	-	-	1338	1:50
75	08110.09547.D70	5351	12	1:10	1:10	BMI304	108	120	535	1:50
76	08110.09547.D70	5351	13	1:20	1:20	BMI304	247	260	268	1:50
77 - Step 1	08110.09547.D70	5351	8	1:5	NA	BMI304	32	40	NA	NA
77 - Final	77 - Step 1	1070	30	1:10	1:50	BMI304	270	300	107	1:50
78 - Step 1	08110.09547.D70	5351	2	1:12	NA	BMI304	22	24	NA	NA
78 - Final	78 - Step 1	446	15	1:20	1:240	BMI304	285	300	22	1:50
79 - Step 1	08110.09547.D70	5351	2	1:20	NA	BMI304	38	40	NA	NA
79 - Final	79 - Step 1	268	12	1:25	1:500	BMI304	288	300	11	1:50
80 - Step 1	08110.09547.D70	5351	2	1:20	NA	BMI304	38	40	NA	NA
80 - Final	80 - Step 1	268	8	1:40	1:800	BMI304	312	320	7	1:50
81	08110.76302.D70	6418	270	1:1	1:1	NA	0	270	6418	1:300
82	Same as QTS 81	6418	-	-	-	-	-	-	6418	1:600
83	Same as QTS 81	6418	-	-	-	-	-	-	6418	1:150
84	08110.76302.D70	6418	12	1:10	1:10	BMI304	108	120	642	1:50
85	08110.76302.D70	6418	5	1:25	1:25	BMI304	120	125	257	1:50
86	08110.76302.D70	6418	7	1:45	1:45	BMI304	308	315	143	1:50
87 - Step 1	08110.76302.D70	6418	4	1:10	NA	BMI304	36	40	NA	NA
87 - Final	87 - Step 1	642	30	1:10	1:100	BMI304	270	300	64	1:50
88 - Step 1	08110.76302.D70	6418	4	1:10	NA	BMI304	36	40	NA	NA
88 - Final	88 - Step 1	642	10	1:30	1:300	BMI304	290	300	21	1:50
89 - Step 1	08110.76302.D70	6418	2	1:20	NA	BMI304	38	40	NA	NA
89 - Final	89 - Step 1	321	10	1:30	1:600	BMI304	290	300	11	1:50
90 - Step 1	08110.76302.D70	6418	4	1:5	NA	BMI304	16	20	NA	NA
90 - Step 2	90 - Step 1	1284	5	1:10	NA	BMI304	45	50	NA	NA
90 - Final	90 - Step 2	128	15	1:24	1:1200	BMI304	345	360	5	1:50

QTS Number	Test Specimen ID	Anti-GP IgG Concentration (ELISA Units/mL) (Before Spike)	Volume of Test Specimen (µL)	Dilution Factor (Spike)	Final Dilution Factor (Spike)	Negative Specimen Identifier (Diluent)	Volume of Negative Specimen Diluent (µL)	Total QTS Volume (µL)	Expected QTS Anti-GP IgG Concentration (ELISA Units/mL)	QTS Starting Dilution
91	08110.19687.D70	7469	300	1:1	1:1	NA	0	300	7469	1:400
92	08110.19687.D70	7469	90	1:3	1:3	BMI304	180	270	2490	1:100
93	Same as QTS 92	7469	-	-	-	-	-	-	2490	1:200
94	Same as QTS 92	7469	-	-	-	-	-	-	2490	1:50
95	08110.19687.D70	7469	20	1:9	1:9	BMI304	160	180	830	1:50
96	08110.19687.D70	7469	10	1:27	1:27	BMI304	260	270	277	1:50
97 - Step 1	08110.19687.D70	7469	2	1:9	NA	BMI304	16	18	NA	NA
97 - Final	97 - Step 1	830	20	1:6	1:54	BMI304	100	120	138	1:50
98 - Step 1	08110.19687.D70	7469	2	1:18	NA	BMI304	34	36	NA	NA
98 - Final	98 - Step 1	415	15	1:20	1:360	BMI304	285	300	21	1:50
99 - Step 1	08110.19687.D70	7469	2	1:20	NA	BMI304	38	40	NA	NA
99 - Final	99 - Step 1	373	4	1:30	1:600	BMI304	116	120	12	1:50
100 - Step 1	08110.19687.D70	7469	2	1:24	NA	BMI304	46	48	NA	NA
100 - Final	100 - Step 1	311	10	1:48	1:1152	BMI304	470	480	6	1:50

Table S6. SUDV Qualification Test Sample Dilutional Linearity Series Generation.

QTS Number	Test Specimen ID	Anti-GP IgG Concentration (ELISA Units/mL) (Before Spike)	Volume of Test Specimen (µL)	Dilution Factor (Spike)	Final Dilution Factor (Spike)	Negative Specimen Identifier (Diluent)	Volume of Negative Specimen Diluent (µL)	Total QTS Volume (µL)	Expected QTS Anti-GP IgG Concentration (ELISA Units/mL)	QTS Starting Dilution
1	FLV1001-100027 Day 78	13143	480	1:1	1:1	NA	0	480	13143	1:700
2	Same as QTS 1	13143	-	-	-	NA	-	-	13143	1:1400
3	Same as QTS 1	13143	-	-	-	NA	-	-	13143	1:350
4	FLV1001-100027 Day 78	13143	160	1:2	1:2	BMI530	160	320	6572	1:300
5	FLV1001-100027 Day 78	13143	40	1:5	1:5	BMI530	160	200	2629	1:100
6	FLV1001-100027 Day 78	13143	32	1:10	1:10	BMI530	288	320	1314	1:100
7	FLV1001-100027 Day 78	13143	10	1:20	1:20	BMI530	190	200	657	1:50
8 - Step 1	FLV1001-100027 Day 78	13143	4	1:20	NA	BMI530	76	80	NA	NA
8 - Final	8 - Step 1	657	16	1:20	1:400	BMI530	304	320	33	1:50
9 - Step 1	FLV1001-100027 Day 78	13143	4	1:20	NA	BMI530	76	80	NA	NA
9 - Final	9 - Step 1	657	6	1:40	1:800	BMI530	234	240	16	1:50
10 - Step 1	FLV1001-100027 Day 78	13143	5	1:16	NA	BMI530	75	80	NA	NA
10 - Final	10 - Step 1	821	3	1:100	1:1600	BMI530	297	300	8	1:50
11	FLV1001-100108 Day 113	5959	200	1:1	1:1	NA	0	200	5959	1:300
12	FLV1001-100108 Day 113	5959	120	1:4	1:4	BMI530	360	480	1490	1:100
13	Same as QTS 12	5959	-	-	-	-	-	-	1490	1:200
14	Same as QTS 12	5959	-	-	-	-	-	-	1490	1:50
15	FLV1001-100108 Day 113	5959	25	1:8	1:8	BMI530	175	200	745	1:50
16	FLV1001-100108 Day 113	5959	30	1:16	1:16	BMI530	450	480	372	1:50
17	FLV1001-100108 Day 113	5959	8	1:30	1:30	BMI530	232	240	199	1:50
18 - Step 1	FLV1001-100108 Day 113	5959	8	1:10	NA	BMI530	72	80	NA	NA
18 - Final	18 - Step 1	596	10	1:20	1:200	BMI530	190	200	30	1:50
19 - Step 1	FLV1001-100108 Day 113	5959	5	1:18	NA	BMI530	85	90	NA	NA
19 - Final	19 - Step 1	331	8	1:25	1:450	BMI530	192	200	13	1:50
20 - Step 1	FLV1001-100108 Day 113	5959	4	1:30	NA	BMI530	116	120	NA	NA
20 - Final	20 - Step 1	199	20	1:25	1:750	BMI530	480	500	8	1:50
21	FLV1001-100041 Day 78	8242	480	1:1	1:1	NA	0	480	8242	1:400
22	Same as QTS 21	8242	-	-	-	-	-	-	8242	1:800
23	Same as QTS 21	8242	-	-	-	-	-	-	8242	1:200
24	FLV1001-100041 Day 78	8242	72	1:5	1:5	BMI530	288	360	1648	1:100

QTS Number	Test Specimen ID	Anti-GP IgG Concentration (ELISA Units/mL) (Before Spike)	Volume of Test Specimen (µL)	Dilution Factor (Spike)	Final Dilution Factor (Spike)	Negative Specimen Identifier (Diluent)	Volume of Negative Specimen Diluent (µL)	Total QTS Volume (µL)	Expected QTS Anti-GP IgG Concentration (ELISA Units/mL)	QTS Starting Dilution
25	FLV1001-100041 Day 78	8242	20	1:10	1:10	BMI530	180	200	824	1:50
26	FLV1001-100041 Day 78	8242	18	1:20	1:20	BMI530	342	360	412	1:50
27 - Step 1	FLV1001-100041 Day 78	8242	9	1:20	NA	BMI530	171	180	NA	NA
27 - Final	27 - Step 1	412	100	1:2	1:40	BMI530	100	200	206	1:50
28 - Step 1	FLV1001-100041 Day 78	8242	9	1:10	NA	BMI530	81	90	NA	NA
28 - Final	28 - Step 1	824	10	1:20	1:200	BMI530	190	200	41	1:50
29 - Step 1	FLV1001-100041 Day 78	8242	6	1:20	NA	BMI530	114	120	NA	NA
29 - Final	29 - Step 1	412	8	1:25	1:500	BMI530	192	200	16	1:50
30 - Step 1	FLV1001-100041 Day 78	8242	4	1:25	NA	BMI530	96	100	NA	NA
30 - Final	30 - Step 1	330	5	1:40	1:1000	BMI530	195	200	8	1:50
31	FLV1001-100075 Day 78	4701	480	1:1	1:1	NA	0	480	4701	1:200
32	Same as QTS 31	4701	-	-	-	-	-	-	4701	1:400
33	Same as QTS 31	4701	-	-	-	-	-	-	4701	1:100
34	FLV1001-100075 Day 78	4701	80	1:3	1:3	BMI530	160	240	1567	1:100
35	FLV1001-100075 Day 78	4701	30	1:9	1:9	BMI530	240	270	522	1:50
36	FLV1001-100075 Day 78	4701	10	1:36	1:36	BMI530	350	360	131	1:50
37 - Step 1	FLV1001-100075 Day 78	4701	20	1:9	NA	BMI530	160	180	NA	NA
37 - Final	37 - Step 1	522	40	1:6	1:54	BMI530	200	240	87	1:50
38 - Step 1	FLV1001-100075 Day 78	4701	12	1:10	NA	BMI530	108	120	NA	NA
38 - Final	38 - Step 1	470	30	1:20	1:200	BMI530	570	600	24	1:50
39 - Step 1	FLV1001-100075 Day 78	4701	12	1:10	NA	BMI530	108	120	NA	NA
39 - Final	39 - Step 1	470	24	1:30	1:300	BMI530	696	720	16	1:50
40 - Step 1	FLV1001-100075 Day 78	4701	12	1:10	NA	BMI530	108	120	NA	NA
40 - Step 2	40 - Step 1	470	12	1:10	NA	BMI530	108	120	NA	NA
40 - Final	40 - Step 2	47	120	1:6	1:600	BMI530	600	720	8	1:50
41	FLV1001-100097 Day 36	1849	400	1:1	1:1	NA	0	400	1849	1:200
42	FLV1001-100097 Day 36	1849	240	1:2	1:2	BMI530	240	480	924	1:100
43	Same as QTS 42	1849	-	-	-	-	-	-	924	1:200
44	Same as QTS 42	1849	-	-	-	-	-	-	924	1:50
45	FLV1001-100097 Day 36	1849	60	1:4	1:4	BMI530	180	240	462	1:50
46	FLV1001-100097 Day 36	1849	45	1:8	1:8	BMI530	315	360	231	1:50

QTS Number	Test Specimen ID	Anti-GP IgG Concentration (ELISA Units/mL) (Before Spike)	Volume of Test Specimen (µL)	Dilution Factor (Spike)	Final Dilution Factor (Spike)	Negative Specimen Identifier (Diluent)	Volume of Negative Specimen Diluent (µL)	Total QTS Volume (µL)	Expected QTS Anti-GP IgG Concentration (ELISA Units/mL)	QTS Starting Dilution
47	FLV1001-100097 Day 36	1849	15	1:16	1:16	BMI530	225	240	116	1:50
48 - Step 1	FLV1001-100097 Day 36	1849	6	1:10	NA	BMI530	54	60	NA	NA
48 - Final	48 - Step 1	185	24	1:20	1:200	BMI530	456	480	9	1:50
49 - Step 1	FLV1001-100097 Day 36	1849	6	1:10	NA	BMI530	54	60	NA	NA
49 - Final	49 - Step 1	185	16	1:15	1:150	BMI530	224	240	12	1:50
50 - Step 1	FLV1001-100097 Day 36	1849	6	1:10	NA	BMI530	54	60	NA	NA
50 - Final	50 - Step 1	185	8	1:30	1:300	BMI530	232	240	6	1:50
51	0740011.18305.D28	1299	400	1:1	1:1	NA	0	400	1299	1:200
52	0740011.18305.D28	1299	330	1:2	1:2	BMI304	330	660	650	1:100
53	Same as QTS 52	1299	-	-	-	-	-	-	650	1:200
54	Same as QTS 52	1299	-	-	-	-	-	-	650	1:50
55	0740011.18305.D28	1299	100	1:4	1:4	BMI304	300	400	325	1:50
56	0740011.18305.D28	1299	75	1:8	1:8	BMI304	525	600	162	1:50
57	0740011.18305.D28	1299	25	1:20	1:20	BMI304	475	500	65	1:50
58 - Step 1	0740011.18305.D28	1299	50	1:3	NA	BMI304	100	150	NA	NA
58 - Final	58 - Step 1	433	60	1:10	1:30	BMI304	540	600	43	1:50
59 - Step 1	0740011.18305.D28	1299	20	1:8	NA	BMI304	140	160	NA	NA
59 - Final	59 - Step 1	162	72	1:10	1:80	BMI304	648	720	16	1:50
60 - Step 1	0740011.18305.D28	1299	15	1:10	NA	BMI304	135	150	NA	NA
60 - Final	60 - Step 1	130	30	1:20	1:200	BMI304	570	600	6	1:50
61	08110.19687.D28	807	480	1:1	NA	NA	0	480	807	1:100
62	Same as QTS 61	807	-	-	-	-	-	-	807	1:200
63	Same as QTS 61	807	-	-	-	-	-	-	807	1:50
64	08110.19687.D28	807	100	1:2	1:2	BMI304	100	200	403	1:50
65	08110.19687.D28	807	50	1:4	1:4	BMI304	150	200	202	1:50
66	08110.19687.D28	807	50	1:8	1:8	BMI304	350	400	101	1:50
67	08110.19687.D28	807	10	1:20	1:20	BMI304	190	200	40	1:50
68 - Step 1	08110.19687.D28	807	16	1:10	NA	BMI304	144	160	NA	NA
68 - Final	68 - Step 1	81	50	1:4	1:40	BMI304	150	200	20	1:50
69 - Step 1	08110.19687.D28	807	30	1:8	NA	BMI304	210	240	NA	NA
69 - Final	69 - Step 1	101	20	1:10	1:80	BMI304	180	200	10	1:50

QTS Number	Test Specimen ID	Anti-GP IgG Concentration (ELISA Units/mL) (Before Spike)	Volume of Test Specimen (µL)	Dilution Factor (Spike)	Final Dilution Factor (Spike)	Negative Specimen Identifier (Diluent)	Volume of Negative Specimen Diluent (µL)	Total QTS Volume (µL)	Expected QTS Anti-GP IgG Concentration (ELISA Units/mL)	QTS Starting Dilution
70 - Step 1	08110.19687.D28	807	16	1:20	NA	BMI304	304	320	NA	NA
70 - Final	70 - Step 1	40	50	1:8	1:160	BMI304	350	400	5	1:50
71	0740011.59335.D63	5273	400	1:1	1:1	NA	0	400	5273	1:700
72	0740011.59335.D63	5273	300	1:4	1:4	BMI304	900	1200	1318	1:100
73	Same as QTS 72	5273	-	-	-	-	-	-	1318	1:200
74	Same as QTS 72	5273	-	-	-	-	-	-	1318	1:50
75	0740011.59335.D63	5273	45	1:10	1:10	BMI304	405	450	527	1:50
76	0740011.59335.D63	5273	27	1:20	1:20	BMI304	513	540	264	1:50
77 - Step 1	0740011.59335.D63	5273	36	1:5	NA	BMI304	144	180	NA	NA
77 - Final	77 - Step 1	1055	54	1:10	1:50	BMI304	486	540	105	1:50
78 - Step 1	0740011.59335.D63	5273	18	1:20	NA	BMI304	342	360	NA	NA
78 - Final	78 - Step 1	264	27	1:20	1:400	BMI304	513	540	13	1:50
79 - Step 1	0740011.59335.D63	5273	15	1:24	NA	BMI304	345	360	NA	NA
79 - Final	79 - Step 1	220	25	1:24	1:576	BMI304	575	600	9	1:50
80 - Step 1	0740011.59335.D63	5273	16	1:30	NA	BMI304	464	480	NA	NA
80 - Final	80 - Step 1	176	18	1:40	1:1200	BMI304	702	720	4	1:50
81	0740011.84569.D70	3358	1200	1:1	1:1	NA	0	1200	3358	1:200
82	Same as QTS 81	3358	-	-	-	-	-	-	3358	1:400
83	Same as QTS 81	3358	-	-	-	-	-	-	3358	1:100
84	0740011.84569.D70	3358	45	1:10	1:10	BMI304	405	450	336	1:50
85	0740011.84569.D70	3358	18	1:25	1:25	BMI304	432	450	134	1:50
86	0740011.84569.D70	3358	12	1:45	1:45	BMI304	528	540	75	1:50
87 - Step 1	0740011.84569.D70	3358	18	1:10	NA	BMI304	162	180	NA	NA
87 - Final	87 - Step 1	336	54	1:10	1:100	BMI304	486	540	34	1:50
88 - Step 1	0740011.84569.D70	3358	36	1:10	NA	BMI304	324	360	NA	NA
88 - Final	88 - Step 1	336	18	1:30	1:300	BMI304	522	540	11	1:50
89 - Step 1	0740011.84569.D70	3358	24	1:20	NA	BMI304	456	480	NA	NA
89 - Final	89 - Step 1	168	24	1:30	1:600	BMI304	696	720	6	1:50
90 - Step 1	0740011.84569.D70	3358	72	1:5	NA	BMI304	288	360	NA	NA
90 - Step 2	90 - Step 1	672	48	1:10	NA	BMI304	432	480	NA	NA
90 - Final	90 - Step 2	67	30	1:24	1:1200	BMI304	690	720	3	1:50

QTS Number	Test Specimen ID	Anti-GP IgG Concentration (ELISA Units/mL) (Before Spike)	Volume of Test Specimen (µL)	Dilution Factor (Spike)	Final Dilution Factor (Spike)	Negative Specimen Identifier (Diluent)	Volume of Negative Specimen Diluent (µL)	Total QTS Volume (µL)	Expected QTS Anti-GP IgG Concentration (ELISA Units/mL)	QTS Starting Dilution
91	0740011.81800.D84	2950	400	1:1	1:1	NA	0	400	2950	1:100
92	0740011.81800.D84	2950	400	1:3	1:3	BMI304	800	1200	983	1:50
93	Same as QTS 92	2950	-	-	-	-	-	-	983	1:100
94	Same as QTS 92	2950	-	-	-	-	-	-	983	1:200
95	0740011.81800.D84	2950	75	1:6	1:6	BMI304	375	450	492	1:50
96	0740011.81800.D84	2950	45	1:12	1:12	BMI304	495	540	246	1:50
97 - Step 1	0740011.81800.D84	2950	30	1:6	NA	BMI304	150	180	NA	NA
97 - Final	97 - Step 1	492	135	1:4	1:24	BMI304	405	540	123	1:50
98 - Step 1	0740011.81800.D84	2950	30	1:12	NA	BMI304	330	360	NA	NA
98 - Final	98 - Step 1	246	54	1:10	1:120	BMI304	486	540	25	1:50
99 - Step 1	0740011.81800.D84	2950	30	1:12	NA	BMI304	330	360	NA	NA
99 - Final	99 - Step 1	246	30	1:20	1:240	BMI304	570	600	12	1:50
100 - Step 1	0740011.81800.D84	2950	24	1:20	NA	BMI304	456	480	NA	NA
100 - Final	100 - Step 1	148	36	1:20	1:400	BMI304	684	720	7	1:50

Table S7. Optimization of Coating Concentration Plate Layout and Optical Density Results – SUDV Data.

rGP Coating Concentration (µg/mL)		Positive Test Sample and Dilution (1:X)										Blank	NC
		1	2	3	4	5	6	7	8	9	10	11	12
		50	100	200	400	800	1600	3200	6400	12,800	25,600	NA	50
**A**	1.0	3.302	2.771	1.958	1.070	0.640	0.323	0.180	0.058	0.04	0.013	0.007	0.068
B	0.5	3.182	2.641	1.816	1.024	0.616	0.267	0.184	0.076	0.056	0.011	0.023	0.021
C	0.4	3.096	2.390	1.666	0.923	0.480	0.250	0.150	0.062	0.030	0.012	0.006	0.022
D	0.3	2.943	2.163	1.438	0.754	0.435	0.185	0.139	0.044	0.055	0.007	0.024	0.012
E	0.2	2.714	1.894	1.258	0.697	0.379	0.174	0.106	0.043	0.026	0.009	0.007	0.016
F	0.1	2.301	1.349	0.875	0.493	0.289	0.130	0.101	0.022	0.029	-0.012	0.039	0.002
G	NA	0.008	-0.003	0.006	-0.002	0.008	-0.003	0.007	-0.002	0.010	-0.005	0.007	0.006
H	NA	0.010	-0.026	0.017	-0.027	0.016	-0.002	0.007	-0.008	0.006	-0.011	0.033	-0.018

NA = Not Applicable; NC = Negative Control

Table S8. Optimization of Coating Concentration Plate Layout and Optical Density Results – MARV Data.

rGP Coating Concentration (µg/mL)		Positive Test Sample and Dilution (1:X)										Blank	NC
		1	2	3	4	5	6	7	8	9	10	11	12
		50	100	200	400	800	1600	3200	6400	12,800	25,600	NA	50
**A**	0.3	3.217	2.409	1.503	0.783	0.481	0.245	0.137	0.067	0.046	0.016	0.013	0.113
B	0.4	3.265	2.543	1.642	0.979	0.586	0.258	0.155	0.065	0.063	0.012	0.025	0.160
C	0.5	3.319	2.687	1.744	0.994	0.548	0.291	0.169	0.081	0.050	0.017	0.010	0.214
D	0.6	3.287	2.739	1.752	1.023	0.591	0.278	0.175	0.079	0.069	0.013	0.020	0.260
E	0.7	3.333	2.726	1.823	1.021	0.594	0.286	0.160	0.076	0.052	0.017	0.013	0.303
F	0.8	3.297	2.754	1.803	1.053	0.586	0.298	0.178	0.080	0.076	0.017	0.028	0.329
G	0.9	3.378	2.824	1.802	1.054	0.585	0.284	0.200	0.077	0.051	0.018	0.011	0.368
H	1.0	3.346	2.817	1.779	1.045	0.597	0.302	0.200	0.091	0.061	0.004	0.035	0.402

NA = Not Applicable; NC = Negative Control

Table S9. Optimization of the Conjugate Dilution– MARV.

	Conjugate Dilution (1:X)	Serum Dilution (1:X)											
		50	100	200	400	800	1,600	3,200	6,400	12,800	25,600	51,200	Blank
		1	2	3	4	5	6	7	8	9	10	11	12
	Positive Serum (MK1)												
A	10,000	3.300	3.090	2.181	1.240	0.703	0.343	0.189	0.083	0.049	0.019	0.024	-0.005
B	12,000	3.250	2.658	1.936	1.055	0.589	0.269	0.177	0.069	0.066	0.018	0.022	-0.008
C	14,000	3.201	2.585	1.651	0.906	0.469	0.248	0.136	0.064	0.040	0.015	0.020	0.008
D	16,000	3.015	2.345	1.020	0.823	0.453	0.207	0.137	0.058	0.064	0.011	0.025	-0.010
E	18,000	3.073	2.231	1.375	0.735	0.410	0.181	0.107	0.049	0.036	0.011	0.020	0.008
F	20,000	2.996	1.874	1.282	0.702	0.369	0.176	0.120	0.044	0.046	0.006	0.034	-0.010
G	22,000	2.917	2.016	1.234	0.698	0.359	0.174	0.099	0.045	0.036	0.009	0.015	0.012
H	Blank	0.007	-0.002	0.022	-0.012	0.015	-0.014	0.022	-0.003	0.016	-0.008	0.029	-0.015
	Negative Serum (BMI530)												
A	10,000	0.056	0.026	0.070	0.077	0.049	-0.002	0.010	-0.003	0.008	-0.004	0.009	-0.004
B	12,000	0.020	0.000	0.023	-0.009	0.023	-0.010	0.029	-0.004	0.029	-0.004	0.017	-0.010
C	14,000	0.048	0.010	0.014	-0.002	0.013	-0.003	0.012	-0.001	0.011	-0.004	0.009	0.006
D	16,000	0.035	0.003	0.024	-0.010	0.020	-0.011	0.026	-0.002	0.034	-0.003	0.015	-0.008
E	18,000	0.034	0.010	0.015	-0.001	0.011	-0.003	0.012	-0.001	0.009	-0.003	0.009	0.005
F	20,000	0.015	-0.003	0.022	-0.013	0.020	-0.011	0.027	-0.006	0.026	-0.005	0.031	-0.009
G	22,000	0.016	0.009	0.014	0.002	0.010	-0.002	0.013	0.002	0.009	-0.002	0.008	0.008
H	Blank	0.009	-0.005	0.022	-0.008	0.015	-0.016	0.025	-0.003	0.020	-0.022	0.038	-0.006
	Binding Ratios (Positive serum OD/Negative serum OD)												
A	10,000	58.929	118.846	31.157	16.104	14.347	-171.500	18.900	-27.667	6.125	-4.750	2.667	1.250
B	12,000	162.500	NA	84.174	-117.222	25.609	-26.900	6.103	-17.250	2.276	-4.500	1.294	0.800
C	14,000	66.688	258.500	117.929	-453.000	36.077	-82.667	11.333	-64.000	3.636	-3.750	2.222	1.333
D	16,000	86.143	781.667	42.500	-82.300	22.650	-18.818	5.269	-29.000	1.882	-3.667	1.667	1.250
E	18,000	90.382	223.100	91.667	-735.000	37.273	-60.333	8.917	-49.000	4.000	-3.667	2.222	1.600
F	20,000	199.733	-624.667	58.273	-54.000	18.450	-16.000	4.444	-7.333	1.769	-1.200	1.097	1.111
G	22,000	182.313	224.000	88.143	349.000	35.900	-87.000	7.615	22.500	4.000	-4.500	1.875	1.500
H	Blank	0.778	0.400	1.000	1.500	1.000	0.875	0.880	1.000	0.800	0.364	0.763	2.500

Table S10. Optimization of the Conjugate Dilution– SUDV.

	Conjugate Dilution (1:X)	Serum Dilution (1:X)											
		50	100	200	400	800	1,600	3,200	6,400	12,800	25,600	51,200	Blank
		1	2	3	4	5	6	7	8	9	10	11	12
	Positive Serum (G26)												
A	6,000	3.241	3.050	2.277	0.992	0.721	0.421	0.230	0.115	0.058	0.027	0.020	0.002
B	7,000	3.139	2.918	2.007	0.915	0.625	0.361	0.222	0.102	0.060	0.022	0.036	-0.011
C	8,000	3.143	2.615	1.868	0.528	0.559	0.335	0.202	0.095	0.054	0.023	0.029	0.005
D	9,000	2.423	2.357	1.515	0.440	0.549	0.277	0.179	0.079	0.060	0.016	0.030	-0.002
E	10,000	3.042	2.266	1.447	0.595	0.510	0.279	0.150	0.076	0.048	0.019	0.020	0.008
F	11,000	3.042	2.326	1.533	0.886	0.539	0.244	0.162	0.068	0.063	0.008	0.026	-0.008
G	12,000	3.025	2.041	1.519	0.896	0.512	0.246	0.153	0.079	0.041	0.018	0.014	0.004
H	Blank	0.006	-0.016	0.022	-0.008	0.019	-0.014	0.028	-0.004	0.018	-0.004	0.003	-0.009
	Negative Serum (BMI529)												
A	6,000	0.019	0.011	0.016	0.001	0.011	0.000	0.009	-0.001	0.008	-0.004	0.006	0.003
B	7,000	0.031	0.006	0.021	-0.004	0.019	-0.005	0.022	-0.001	0.021	-0.003	0.024	-0.006
C	8,000	0.030	0.014	0.015	0.000	0.010	-0.001	0.009	0.000	0.009	-0.002	0.003	0.006
D	9,000	0.029	-0.008	0.027	-0.006	0.029	-0.005	0.020	-0.005	0.021	-0.008	0.020	-0.004
E	10,000	0.036	0.011	0.015	-0.001	0.010	0.000	0.010	-0.001	0.010	-0.003	0.010	0.005
F	11,000	0.039	-0.005	0.027	-0.009	0.028	-0.011	0.029	-0.002	0.025	-0.015	0.036	-0.011
G	12,000	0.025	0.013	0.015	0.002	0.006	0.000	0.008	0.001	0.003	-0.003	0.006	0.008
H	Blank	0.006	-0.006	0.008	0.005	0.017	-0.005	0.028	-0.005	0.005	0.001	0.003	-0.012
	Binding Ratios (Positive serum OD/Negative serum OD)												
A	6,000	170.579	277.273	142.313	992.000	65.545	NA	25.556	-115.000	7.250	-6.750	3.333	0.667
B	7,000	101.258	486.333	95.571	-228.750	32.895	-72.200	10.091	-102.000	2.857	-7.333	1.500	1.833
C	8,000	104.767	186.786	124.533	#DIV/0!	55.900	-335.000	22.444	NA	6.000	-11.500	9.667	0.833
D	9,000	83.552	-294.625	56.111	-73.333	18.931	-55.400	8.950	-15.800	2.857	-2.000	1.500	0.500
E	10,000	84.500	206.000	96.467	-595.000	51.000	NA	15.000	-76.000	4.800	-6.333	2.000	1.600
F	11,000	78.000	-465.200	56.778	-98.444	19.250	-22.182	5.586	-34.000	2.520	-0.533	0.722	0.727
G	12,000	121.000	157.000	101.267	448.000	85.333	NA	19.125	79.000	13.667	-6.000	2.333	0.500
H	Blank	1.000	2.667	2.750	-1.600	1.118	2.800	1.000	0.800	3.600	-4.000	1.000	0.750

Table S11. Potential Outliers Identified from Mixed Analysis of Variance Model – Human MARV.

Sample	Observed Value (ELISA Units/mL)	Model-Predicted Value (ELISA Units/mL)	Residual
QTS 2	3665.24	1496.82	5.12
QTS 26	172.52	75.38	4.63
QTS 32	2142.64	4043.12	-3.70
QTS 40	1.79	4.00	-4.79
QTS 48	25.81	10.70	4.97

Outliers were included in statistical analysis.

Table S12. Potential Outliers Identified from Mixed Analysis of Variance Model – NHP MARV.

Sample	Observed Value (ELISA Units/mL)	Model-Predicted Value (ELISA Units/mL)	Residual
QTS 88	6.83	11.09	-3.52
QTS 98	5.44	12.49	-4.78

Outliers were included in statistical analysis.

Table S13. Potential Outliers Identified from Mixed Analysis of Variance Model – Human SUDV.

Sample	Observed Value (ELISA Units/mL)	Model-Predicted Value (ELISA Units/mL)	Residual
QTS 15*	0.00	287.43	-12.50
QTS 16*	1,287.89	489.21	5.84
QTS 18*	0.00	16.91	-6.25
QTS 30^	4.55	11.67	-7.77
QTS 30^	34.69	13.53	7.77
QTS 38^	10.44	19.58	-3.97

^Outliers were included in statistical analysis.
*Outliers were excluded from statistical analysis.

Table S14. Potential Outliers Identified from Mixed Analysis of Variance Model – NHP SUDV.

Sample	Observed Value (ELISA Units/mL)	Model-Predicted Value (ELISA Units/mL)	Residual
QTS 90	3.03	5.99	-5.15
QTS 98	138.89	72.83	4.84
QTS 80	0.00	6.34	-10.07
QTS 90	0.00	6.07	-9.49

Outliers were included in statistical analysis.

Table S15. Geometric Mean Concentration and Percent Coefficient of Variation (%CV) by Dilutional Linearity Test Samples (QTS 1-50) – Human and MARV.

FLV1001-100023 Day 71				FLV1001-100068 Day 71				FLV1001-100074 Day 71			
QTS	N	Geometric Mean Concentration (ELISA Units/mL)	%CV	QTS	N	Geometric Mean Concentration (ELISA Units/mL)	%CV	QTS	N	Geometric Mean Concentration (ELISA Units/mL)	%CV
1	9	1226.80	22.7	11	8	1746.33	20.5	21	8	2328.23	20.8
2	10	1351.80	49.2	12	8	807.88	23.4	22	8	2351.00	18.1
3	9	1332.98	17.7	13	8	805.66	35.4	23	8	2407.99	40.6
4	17	628.59	26.9	14	8	841.55	25.5	24	18	759.08	36.3
5	9	219.13	23.5	15	8	376.67	26.4	25	8	473.33	22.0
6	18	146.97	29.7	16	21	186.95	32.8	26	19	83.32	36.2
7	9	65.18	19.3	17	8	101.42#	27.6	27	8	32.97	40.0
8	20	12.98#	34.1	18	8	8.53	118.7*	28	8	10.58#	40.9
9	9	5.86	138.9*	19	7	2.49	172.8*	29	8	1.98	102.0*
10	9	3.88	235.4*	20	16	1.14	53.1*	30	8	1.14	37.1

FLV1001-100014 Day 71				FLV1001-100073 Day 71			
QTS	N	Geometric Mean Concentration (ELISA Units/mL)	%CV	QTS	N	Geometric Mean Concentration (ELISA Units/mL)	%CV
31	8	5232.12^	19.9	41	17	1291.02	19.4
32	8	4287.67	45.1	42	8	605.60	18.4
33	8	4959.56	18.1	43	8	632.11	18.2
34	8	1436.65	20.3	44	8	678.60	7.8
35	8	503.91	19.5	45	8	313.42	12.8
36	16	161.58	29.7	46	16	160.19	16.7
37	8	76.69#	25.1	47	8	80.28#	17.4
38	8	9.04	121.4*	48	17	5.85	179.2*
39	8	4.83	136.3*	49	8	1.69	132.1*
40	16	1.86	105.8*	50	8	1.26	74.3*

Shaded cells reflect test samples with at least 95% detection rate (≥LOD).

*%CV not acceptable (>50%).

#Geometric mean reportable value at the lowest-concentration sample with acceptable precision. Preliminary LLOQ is the median of these values across the five parent samples (76.69).

^Preliminary ULOQ.

Table S16. Geometric Mean Concentration and Percent Coefficient of Variation (%CV) by Dilutional Linearity Test Samples (QTS 51-100) – NHP and MARV.

08110.28552.D70				08110.67181.D70				08110.09547.D70			
QTS	N	Geometric Mean Concentration (ELISA Units/mL)	%CV	QTS	N	Geometric Mean Concentration (ELISA Units/mL)	%CV	QTS	N	Geometric Mean Concentration (ELISA Units/mL)	%CV
51	8	4118.17	25.5	61	8	4421.63	34.9	71	8	6096.25	6.3
52	8	477.66	15.5	62	8	4928.86	32.3	72	8	1564.86	14.7
53	8	475.71	31.1	63	8	4919.85	38.2	73	8	1644.12	18.2
54	16	468.88	30.1	64	8	585.86	28.4	74	17	1542.65	21.0
55	8	178.91	33.2	65	8	220.37	18.8	75	8	578.73	34.1
56	16	100.79	28.6	66	17	106.64	21.7	76	16	289.08	16.3
57	8	33.20#	24.6	67	8	58.98#	27.1	77	8	108.52	20.7
58	17	12.33	327.8*	68	8	13.02	146.1*	78	8	20.42#	28.0
59	8	3.65	157.1*	69	8	12.76	39.7	79	8	4.46	212.9*
60	8	1.30	82.4*	70	17	1.50	91.9*	80	8	0.00	--

08110.76302.D70				08110.19687.D70			
QTS	N	Geometric Mean Concentration (ELISA Units/mL)	%CV	QTS	N	Geometric Mean Concentration (ELISA Units/mL)	%CV
81	8	5449.96	24.7	91	16	6786.82	26.6
82	8	6084.30	41.3	92	8	2032.97	28.0
83	8	6898.26^	46.1	93	8	2444.31	20.7
84	8	608.38	26.6	94	8	2189.53	25.5
85	8	238.26	26.8	95	8	715.36	28.7
86	16	142.08	20.9	96	16	217.13	15.8
87	8	56.88	18.3	97	8	84.59	20.6
88	8	14.15#	41.9	98	19	17.13#	35.9
89	7	5.04	167.2*	99	7	4.68	151.7*
90	16	1.25	84.0*	100	8	0.00	--

Shaded cells reflect test samples with at least 95% detection rate (≥LOD).
*%CV not acceptable (>50%).
**%CV not acceptable (>50%) but test sample was included in overall precision/linearity analyses since QTS were near middle of assay range.
--All observations were zero and thus there was no variability.
#Geometric mean reportable value at the lowest-concentration sample with acceptable precision. Preliminary LLOQ is the median of these values across the five parent samples (20.42).
^Preliminary ULOQ

Table S17. Geometric Mean Concentration and Percent Coefficient of Variation (%CV) by Dilutional Linearity Test Samples (QTS 1-50) – Human and SUDV.

FLV1001-100027 Day 78				FLV1001-100108 Day 113				FLV1001-100041 Day 78			
QTS	N	Geometric Mean Concentration (ELISA Units/mL)	%CV	QTS	N	Geometric Mean Concentration (ELISA Units/mL)	%CV	QTS	N	Geometric Mean Concentration (ELISA Units/mL)	%CV
1	8	11,542.60^	20.6	11	8	7,108.14	15.4	21	8	7,260.71	14.9
2	8	10,938.70	18.7	12	8	1,774.80	17.6	22	8	7,463.71	16.1
3	8	10,846.08	23.6	13	8	1,887.89	22.3	23	8	7,613.38	22.3
4	16	5,788.16	18.5	14	8	1,723.56	23.2	24	16	1,446.39	23.6
5	8	1,984.42	18.1	15	7	811.01	14.8	25	8	725.47	17.9
6	16	1,095.72	23.1	16	15	388.53	15.1	26	17	341.01	16.7
7	8	437.27	13.0	17	8	231.45	24.7	27	7	158.37	15.4
8	17	22.70	19.6	18	7	30.79	26.7	28	6	31.26	20.7
9	8	10.82#	35.4	19	8	12.80#	34.2	29	7	11.56#	26.7
10	8	2.14	168.0*	20	16	6.09	98.5*	30	8	1.88	231.1*

FLV1001-100075 Day 78				FLV1001-100097 Day 36			
QTS	N	Geometric Mean Concentration (ELISA Units/mL)	%CV	QTS	N	Geometric Mean Concentration (ELISA Units/mL)	%CV
31	9	5,137.20	30.1	41	16	1,558.09	49.3
32	9	4,840.60	11.7	42	8	694.52	25.2
33	8	5,127.31	22.7	43	8	752.50	45.1
34	8	1,684.33	11.5	44	8	778.03	15.8
35	8	542.77	11.9	45	8	369.31	22.4
36	16	111.71	20.3	46	16	178.68	15.8
37	8	71.93	12.5	47	8	80.28#	17.3
38	8	20.87	51.1*	48	16	2.66	145.4*
39	8	11.32#	25.7	49	8	7.72	47.2
40	16	4.05	91.3*	50	8	1.22	62.5*

Shaded cells reflect test samples with at least 95% detection rate.

*%CV not acceptable (>50%).

#Geometric mean reportable value at the lowest-concentration sample with acceptable precision. Preliminary LLOQ is the median of these values across the five parent samples (11.56).

^Preliminary ULOQ (11,542.60).

Table S18. Geometric Mean Concentration and Percent Coefficient of Variation (%CV) by Dilutional Linearity Test Samples (QTS 51-100) – NHP and SUDV.

0740011.18305.D28				08110.19687.D28				0740011.59335.D63			
QTS	N	Geometric Mean Concentration (ELISA Units/mL)	%CV	QTS	N	Geometric Mean Concentration (ELISA Units/mL)	%CV	QTS	N	Geometric Mean Concentration (ELISA Units/mL)	%CV
51	7	4,743.80	24.9	61	7	2,594.15	36.3	71	9	17,199.09^	25.9
52	7	2,015.39	33.8	62	8	2,624.81	24.0	72	8	4,326.81	23.8
53	7	2,151.38	28.3	63	7	2,942.61	20.9	73	8	3,982.17	17.9
54	13	2,030.36	17.1	64	8	1,359.63	24.9	74	6	4,422.19	34.8
55	7	1,028.07	22.7	65	9	642.06	28.8	75	7	1,621.92	26.7
56	13	513.26	19.4	66	16	310.17	9.2	76	16	870.81	20.6
57	7	178.81	32.8	67	8	123.66	29.9	77	8	278.84	17.7
58	15	129.07	15.6	68	8	57.39	22.4	78	8	36.91	36.0
59	7	47.60	22.0	69	8	28.94	26.5	79	8	25.95#	36.7
60	6	18.10#	33.8	70	14	14.89#	36.9	80	8	9.71	122.4*

0740011.84569.D70				0740011.81800.D84			
QTS	N	Geometric Mean Concentration (ELISA Units/mL)	%CV	QTS	N	Geometric Mean Concentration (ELISA Units/mL)	%CV
81	7	11,564.66	23.5	91	7	14,489.14	37.8
82	8	9,296.83	24.7	92	8	3,760.33	26.7
83	3	10,324.04	16.0	93	9	3,611.04	24.0
84	8	1,021.03	23.9	94	8	3,450.20	29.5
85	8	405.69	18.1	95	9	1,812.92	18.2
86	16	192.64	28.7	96	17	825.76	21.8
87	8	91.32	30.3	97	8	433.95	15.2
88	8	30.23	33.0	98	15	81.28	22.1
89	8	18.55#	29.1	99	9	34.77	21.2
90	16	7.28	81.5*	100	9	23.00#	27.0

Shaded cells reflect test samples with at least 95% detection rate.

*%CV not acceptable (>50%).

#Geometric mean reportable value at the lowest-concentration sample with acceptable precision. Preliminary LLOQ is the median of these values across the five parent samples (18.55).

^Preliminary ULOQ (17.199.09).

Table S19. Specificity Results – Human and MARV.

Test Sample	Geometric Mean Across Days/Operators (ELISA Units/mL)				Ratio of Geometric Means Mock to Antigen		
	**Mock**	**rGP - 25 µg/mL**	**rGP - 10 µg/mL**	**CMV - 25 µg/mL**	**Mock / rGP25**	**Mock / rGP10**	**Mock / CMV**
FLV1001-100041 Day 92	1348.82	4.21	9.97	1020.32	320.32	135.30	1.32
FLV1001-100063 Day 71	1050.90	1.60	12.59	999.96	657.15	83.48	1.05
FLV1001-100027 Day 92	873.75	3.52	35.21	721.60	248.30	24.81	1.21
FLV1001-100102 Day 180	574.24	0.00	5.64	595.36	--	101.86	0.96
FLV1001-100005 Day 92	689.46	2.89	18.40	614.48	238.58	37.46	1.12
**Mean**					**366.09**	**76.58**	**1.13**
**90% Lower Bound**					**133.77**	**33.04**	**1.00**
**90% Upper Bound**					**598.41**	**120.13**	**1.27**

-- Geometric mean for antigen was zero, thus ratio could not be calculated.

Table S20. Specificity Results – NHP and MARV.

Test Sample	Geometric Mean Across Days/Operators (ELISA Units/mL)				Ratio of Geometric Means Mock to Antigen		
	**Mock**	**rGP - 25 µg/mL**	**rGP - 10 µg/mL**	**CMV - 25 µg/mL**	**Mock / rGP25**	**Mock / rGP10**	**Mock / CMV**
08110.39648.D88	669.67	1.20	18.47	595.86	559.36	36.25	1.12
08110.43742.D77	1459.87	5.67	74.02	1367.94	257.40	19.72	1.07
08110.52555.D70	2537.22	133.87	292.69	2369.40	18.95	8.67	1.07
08110.67181.D77	2507.72	175.27	241.87	2566.08	14.31	10.37	0.98
08110.92454.D77	2138.32	93.63	256.04	2099.94	22.84	8.35	1.02
**Mean**					**174.57**	**16.67**	**1.05**
**90% Lower Bound**					**0.00^**	**5.33**	**1.00**
**90% Upper Bound**					**402.12**	**28.01**	**1.10**

^ Negative lower bound replaced with zero.

Table S21. Specificity Results – Human and SUDV.

Test Sample	Geometric Mean Across Days/Operators (ELISA Units/mL)				Ratio of Geometric Means Mock to Antigen		
	Mock	rGP - 25 µg/mL	rGP - 10 µg/mL	CMV - 25 µg/mL	Mock / rGP25	Mock / rGP10	Mock / CMV
FLV1001-100027 Day 57	1,839.59	817.50	916.27	1,778.98	2.25	2.01	1.03
FLV1001-100066 Day 78<br>FLV1001-100116 Day 36	1,365.14	181.20	264.01	1,277.97	7.53	5.17	1.07
FLV1001-100103 Day 36	957.05	337.60	374.65	952.70	2.83	2.55	1.00
FLV1001-100108 Day 92<br>FLV1001-100090 Day 57<br>FLV1001-100111 Day 36	745.21	284.61	326.53	702.76	2.62	2.28	1.06
FLV1001-100108 Day 15	462.53	101.13	149.74	315.59	4.57	3.09	1.47
**Mean**					**3.96**	**3.02**	**1.13**
**90% Lower Bound**					**1.88**	**1.81**	**0.94**
**90% Upper Bound**					**6.05**	**4.23**	**1.31**

Table S22. Specificity Results – NHP and SUDV.

Test Sample	Geometric Mean Across Days/Operators (ELISA Units/mL)				Ratio of Geometric Means Mock to Antigen		
	Mock	rGP - 25 µg/mL	rGP - 10 µg/mL	CMV - 25 µg/mL	Mock / rGP25	Mock / rGP10	Mock / CMV
080100.48343.D63	11,020.49	560.75	1,219.26	8,396.05	19.65	9.04	1.31
0740011.18305.D14	5,995.21	3,191.48	3,882.16	5,458.33	1.88	1.54	1.10
0740011.42672.D42	2,391.78	460.99	723.67 (779.13)*	2,385.88	5.19	3.31	1.00
0740011.81800.D35	2,311.82	--	733.98	2,332.21	--	3.15	0.99
0740011.78934.D42	1,390.23	179.83	379.08	1,353.66	7.73	3.67	1.03
**Mean**					**8.61**	**4.14**	**1.09**
**90% Lower Bound**					**0.00^**	**1.42**	**0.96**
**90% Upper Bound**					**17.72**	**6.86**	**1.21**

-- No passing observations were available for the antigen.

^ Negative lower bound replaced with zero.

Table S23. PsVNA Variability Estimates Obtained from Variance Components from Mixed Analysis of Variance Model.

Variance Component	% CV for $PsVNT_{50}$				% CV for $PsVNT_{80}$			
	MARV		SUDV		MARV		SUDV	
	Human	NHP	Human	NHP	Human	NHP	Human	NHP
Operator	16.9	0.0	0.0	0.0	9.5	0.0	0.0	4.7
Day	6.3	8.0	0.0	13.3	11.5	15.2	0.0	10.3
Plate	6.9	28.4	21.3	6.6	5.8	23.5	15.4	6.1
Intermediate Precision (Sum of Operator, Day, and Plate)	19.4	29.6	21.3	14.8	16.1	28.2	15.4	12.9
Residual (Repeatability)	21.5	22.6	46.4	25.1	27.7	26.1	43.1	19.3
Total Assay Variability	29.2	37.8	52.0	29.4	32.4	39.1	46.3	23.3
Total Assay Variability Range Among Surrogate Test Samples	15.4 – 46.1	26.5 – 54.8	34.2 – 59.1	23.4 – 34.7	17.3 – 56.6	21.7 – 64.2	36.6 – 59.4	13.8 – 30.1

Table S24. Human MARV PsVNA Control Acceptance Criteria.

Control	Parameter	N	Mean	Standard Deviation	Acceptance Criteria
Negative (BMI547)	$PsVNT_{50}$	60	<LOD (4)	-	<LOD (no dilution should result in a percent neutralization of 50% or higher)
	$PsVNT_{80}$	60	<LOD (4)	-	
Positive (MARV-Pos-110719)	$PsVNT_{50}$	43	1159	401	356 – 1962*
	$PsVNT_{80}$	43	263	81	101 – 424*
Cell Culture	Average RLU	80	260	60	100 – 600^
PsV	Average RLU	80	1.98×10^6	3.99×10^5	1,000,000 – 3,500,000^

*Range or values shown = mean +/- 2 standard deviations.

^Ranges provided for RLU based acceptance criteria were expanded beyond the mean +/- 2 standard to account for variation among cell passages, maintain a minimum RLU value for the PsV control, and maintain consistency with assays using the same MARV PsV.

Table S25. Human SUDV PsVNA Control Acceptance Criteria.

Control	Parameter	N	Mean	Standard Deviation	Acceptance Criteria
Negative (BMI547)	$PsVNT_{50}$	60	<LOD (4)	-	<LOD (no dilution should result in a percent neutralization of 50% or higher)
	$PsVNT_{80}$	60	<LOD (4)	-	
Positive (BMISUDV104)	$PsVNT_{50}$	45	307	189	20 – 686*
	$PsVNT_{80}$	45	86	37	20 – 159*
Cell Culture	Average RLU	80	249	55	100 - 600^
PsV	Average RLU	80	2.23×10^5	1.07×10^5	45,000 – 500,000^

*Range or values shown = mean +/- 2 standard deviations. Lower bound of the positive control acceptance criteria was set at 20 to ensure that positive controls have neutralization titers above values that could be interpreted as close to the theoretical LOD (e.g., $PsVNT_{50}$ or $PsVNT_{80}$ between 8 and 16).
^Ranges provided for RLU based acceptance criteria were expanded beyond the mean +/- 2 standard to account for variation among cell passages and to maintain a minimum RLU value for the PsV control.

Table S26. NHP MARV PsVNA Control Acceptance Criteria.

Control	Parameter	N	Mean	Standard Deviation	Acceptance Criteria
Negative (BMI289)	$PsVNT_{50}$	40	<LOD (10)	-	<LOD (no dilution should result in a percent neutralization of 50% or higher)
	$PsVNT_{80}$	40	<LOD (10)	-	
Positive (022620-NHP)	$PsVNT_{50}$	37	935	346	243 – 1627*
	$PsVNT_{80}$	37	264	84	96 – 432*
Cell Culture	Average RLU	80	249	147	100 - 600^
PsV	Average RLU	80	2.23×10^6	4.34×10^5	1,000,000 – 3,500,000^

*Range or values shown = mean +/- 2 standard deviations. The lower bound of the Cell Culture acceptance criteria was set at 100 since a cell culture control value below 100 has never been observed.
^Ranges provided for RLU based acceptance criteria were expanded beyond the mean +/- 2 standard to account for variation among cell passages and to maintain a minimum RLU value for the PsV control.

Table S27. NHP SUDV PSVNA Control Acceptance Criteria.

Control	Parameter	N	Mean	Standard Deviation	Acceptance Criteria
Negative (BMI300)	$PsVNT_{50}$	39	<LOD (10)	-	<LOD (no dilution should result in a percent neutralization of 50% or higher)
	$PsVNT_{80}$	39	<LOD (10)	-	
Positive (NHP-SUDV-PC)	$PsVNT_{50}$	38	2440	839	762 – 4119*
	$PsVNT_{80}$	38	1052	244	563 – 1541*
Cell Culture	Average RLU	80	239	38	100 - 600^
PsV	Average RLU	39	$8.56x10^5$	$1.89x10^5$	45,000 – 1,500,000^

*Range or values shown = mean +/- 2 standard deviations.
^Ranges provided for RLU based acceptance criteria were expanded beyond the mean +/- 2 standard deviations to include the range of RLU values observed during development and characterization efforts. This expansion accounted for variation among cell passages and to maintain a minimum RLU value for the PsV control.

Table S28. PsVNA Sample Acceptance Criteria.

Assay	Parameter	Acceptance Criteria*	Notes
Human MARV	$PsVNT_{50}$	% CV ≤ 40	Test samples that include replicates with values <LOD or 1 to 3-fold the LOD will not be subjected to the %CV value acceptance criteria (e.g., $PsVNT_{50}$ or $PsVNT_{80}$ between 8 and 24).
	$PsVNT_{80}$	% CV ≤ 45	
Human SUDV	$PsVNT_{50}$	% CV ≤ 60	
	$PsVNT_{80}$	% CV ≤ 60	
NHP MARV	$PsVNT_{50}$	% CV ≤ 55	Test samples that include replicates with values <LOD or 1 to 3-fold the LOD will not be subjected to the %CV value acceptance criteria (e.g., $PsVNT_{50}$ or $PsVNT_{80}$ between 20 and 60).
	$PsVNT_{80}$	% CV ≤ 55	
NHP SUDV	$PsVNT_{50}$	% CV ≤ 35	
	$PsVNT_{80}$	% CV ≤ 35	

*Based on Total Assay Variability.
